# Activation of TRPV4 promotes osteoclasts differentiation

**DOI:** 10.1186/ar3643

**Published:** 2012-02-09

**Authors:** Ritsuko Masuyama

**Affiliations:** 1 Department of Cell Biology, Nagasaki University Graduate School of Biomedical Sciences, Nagasaki, Japan

## 

Osteoclast differentiation is critically dependent on cellular calcium (Ca^2+^) signaling. Intracellular Ca^2+ ^concentration ([Ca^2+^]_i_) is regulated by two flux pathways; Ca^2+ ^oscillations evoked by the release of Ca^2+ ^from the endoplasmic reticulum, and/or Ca^2+ ^entry from the extracellular fluid. The latter is carried out by the plasmamembrane localized Ca^2+ ^permeable channel such as "transient receptor potentials (Trps)". Trpv4-deficient mice show an increased bone mass due to impaired osteoclast maturation, because Trpv4 mediates Ca^2+ ^influx at the late stage of osteoclast differentiation and hereby regulates Ca^2+ ^signaling [1]. Furthermore, substitutions of amino acids R616Q/V620I of Trpv4 have been discovered as gain of function mutations resulting in increased Ca^2+ ^transport [2]. Since the region of these substitutions at the trans-membrane pore domain is perfectly conserved between species, we created a mutant of the mouse Trpv4 (Trpv4^R616Q/V620I^) and characterized it on Ca^2+ ^signaling especially in the occurrences of oscillations at the initial step of osteoclast differentiation.

Intact Trpv4 and Trpv4^R616Q/V620I ^were equally transduced by retroviral infection into bone marrow derived hematopoietic cells isolated from WT mice, and mock-transfection was used as control. The resorptive activity was significantly increased in Trpv4^R616Q/V620I^-expressing osteoclasts when treated with RANKL for 7 days, associating increased NFATc1 and calcitonin receptor mRNA expression. Noteworthy, the expression of these differentiation markers was already elevated in Trpv4^R616Q/V620I ^cells before RANKL treatment, suggesting that the activation of Trpv4 advances osteoclast differentiation through Ca^2+^-NFATc1 pathway. Accordingly, basal [Ca^2+^]_i_, analyzed in progenitor cells treated with RANKL for 24 hr, increased 2 fold in intact Trpv4 (p < 0.05) and 3 fold in Trpv4^R616Q/V620I ^(p < 0.01) compared to controls. Although spontaneous Ca^2+ ^oscillations were absent in control progenitor cells, Trpv4^R616Q/V620I ^progenitor cells already displayed irregular oscillatory pattern.

In summary, our findings provide evidences that the activation of Ca^2+ ^permeable channel supports Ca^2+ ^oscillations in progenitor cells and therefore promotes the potential of osteoclast differentiation.
